# A National Survey on Current Practices in Management of The Third Stage of Labor in Second Trimester Deliveries

**DOI:** 10.21203/rs.3.rs-8339323/v1

**Published:** 2026-01-19

**Authors:** Soumya Gogia, Alexandra Sundermann, Ivana Thompson

**Affiliations:** Baylor College of Medicine; Vanderbilt University Medical Center; University of Washington Medical Center

**Keywords:** placenta, delivery, third stage of labor, survey

## Abstract

**Background:**

Retained placenta complicates roughly 21–30% of second trimester deliveries. Literature and clinical guidelines regarding management of the third stage of labor are limited, leading to a wide variation in practice. The purpose of this study is to clarify providers’ current management practices and their supporting rationale.

**Methods:**

A cross-sectional survey was administered to obstetric providers who have managed retained placenta following second trimester deliveries in the last five years via two national listservs of obstetric providers. Descriptive statistics were used to characterize provider characteristics and practices for managing the third stage of labor in second trimester deliveries, including sequence of interventions, medication selection, and time allotted for expectant management.

**Results:**

Of the 113 survey participants, most respondents were OB/Gyn Generalists practicing in urban academic centers. Sixty-seven percent of respondents reported their institution did not have a protocol for managing the third stage of labor in second trimester deliveries. Half of providers reported medical management as first line (53%), with oxytocin and misoprostol being the most commonly used medications. Forty-two percent of participants elected for expectant management as first line, with a median of 120 minutes in duration (inter-quartile range: 30, 240 minutes). Most providers reported that their current practice was either learned practice from a mentor, based on clinical guidelines/scientific literature, or based on clinical scenario.

**Conclusions:**

Management practices for the third stage of labor in second trimester deliveries vary widely across providers, particularly regarding medication selection and duration of expectant management. Many providers reported practice preferences that do not align with extremely limited existing data or clinical guidelines.

## Introduction

Retained placenta complicates roughly 21–30% of second trimester inductions.^1, 2^ The high incidence of retained placenta after second trimester inductions is an area of clinical concern as retained placenta serves as a cause of maternal morbidity after second trimester deliveries. Traditionally, this complication has been managed by instrument removal and curettage, which can be associated with hemorrhage, infection, and uterine perforation.^1^ At this time, the only existing clinical guidance which specifically addresses retained placenta following second trimester deliveries is the Society for Family Planning’s clinical recommendations regarding medication abortion in the second trimester, which advises expectant management up to 4 hours after fetal expulsion for placental delivery and which notes that administration of oxytocin 10 units may be considered.^3^The guideline notes the suggested four-hour limit is based on the need to expedite hospital discharge, and not based on morbidity and mortality outcomes.^3^ While some studies recommend that surgical removal be pursued after 2 hours of expectant management,^4,5,6^ others have found no increase in risk as compared to surgical management when retained placenta is managed expectantly up to 10 hours.^2^ Further, there is heterogeneity in recommendations for optimal medication selection for management of the third stage of labor in second trimester deliveries. Two randomized control trials identified intramuscular 15-methyl prostaglandin F2a as most effective for managing the third stage of labor, yet the use represented in these trials is not routine at many institutions.^6,7^

The paucity of studies regarding optimal management of the third stage of labor in second trimester deliveries creates the opportunity for wide variation in clinical practice. The goal of this study was to elucidate current practices of providers who routinely manage second trimester deliveries and to understand underlying rationale in management decisions.

## Methods

We conducted a cross-sectional study using an internet-based survey regarding obstetric providers’ perspectives and practices for managing the third stage of labor in second trimester deliveries. This survey complies with CHERRIES reporting guidelines and was approved by our university’s IRB.

The survey was distributed via two email listservs, one addressed to Obstetrics & Gynecology program directors with a request to send to trainees and their departments as well as one to Ryan program directors. At the time of this study, the Kenneth J. Ryan Residency Training Program in Abortion and Family Planning supported abortion and contraception training in 109 of 302 ACGME-accredited obstetrics and gynecology residency programs in 37 states. Given the Ryan program’s focus on abortion training and education, it was thought that a large population of providers who assist in performing second trimester deliveries would be captured through this listserv. Two requests for survey participation were sent to each listserv, one in September 2022 and a follow up request in October 2022. Our survey invitation stated that our intended goal was to clarify current practices in managing retained placenta following second trimester delivery given the lack of current clinical guidelines on this topic and potential for significant morbidity from this complication. Participation invitations specified the survey was designed to take approximately 10–15 minutes to complete. Participants were informed that their email addresses would be collected and stored in a redcap database only to ensure no duplicate entries had been collected, and that this information would be deleted once duplicate entries were removed. Providers were eligible to participate if they reported managing a retained placenta following second trimester deliveries in the past five years.

Questions were developed de novo by the research team, which included a board-certified Complex Family Planning specialist at our institution and biostatisticians (Appendix 2). The first survey question confirmed participant eligibility by confirming provider management of a retained placenta following second trimester delivery within the past 5 years. If the participant did not meet eligibility criteria, they were instructed to terminate the survey. Participants provided demographic and practice information including provider type, level of training, practice setting, and geographic region. We also queried whether participants’ institutions had a specific protocol for the management of the third stage of labor following deliveries in general.

The next section of the survey focused on provider preferences for managing the third stage of labor in second trimester deliveries. Participants were first asked to rank in chronological order their preference of management options for the third stage of labor including expectant management, medication management, surgical management, or other. They were further asked to describe if “other” was selected and to give a specific time frame if they allow for expectant management. Participants also ranked their preferences for medication selection and factors that influenced their rationale for management preferences.

We collected and managed study data using REDCap (Research Electronic Data Capture), a secure, web-based software platform designed to support data capture for research studies. All statistical analyses were performed using Stata (Version 18.0, StataCorp, College Station, TX). Information regarding provider characteristics and survey responses were presented as counts and proportions. A Sankey diagram was utilized to demonstrate the progression of management preferences. Responses for time permitted for expectant management were not normally distributed and were reported using median and inter-quartile range (IQR). We used the Wilcoxon rank-sum test to assess for differences in reported time allotted for expectant management between providers who chose expectant management as first-line management and those who did not. Differences in providers’ preference for first-line management method by provider characteristic was assessed using chi-squared tests.

## Results

One hundred and thirteen providers responded to the survey, primarily consisting of OB/Gyn Generalist providers (81%), followed by Maternal Fetal Medicine specialists (12%), and certified nurse midwives (12%). Most providers had completed their training by time of participation (69%) and practiced at an academic hospital (91%) (Table 1). The majority (67%) of providers practiced at a hospital that did have a specific protocol for management of the third stage of labor.

**Figure 1:**
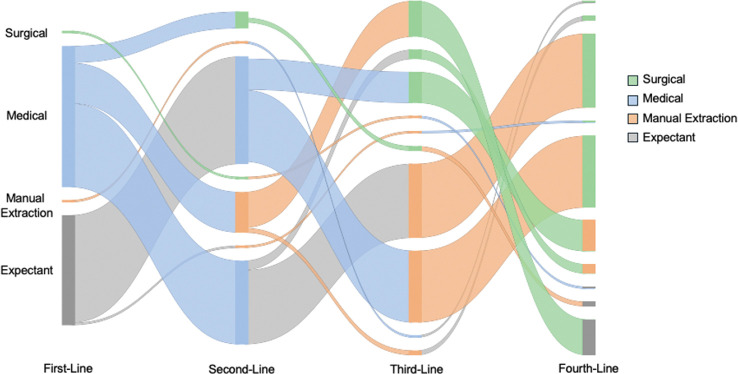
Providers’ preferences for sequence of first-, second-, third-, and fourth-line management of the third stage of labor in second trimester deliveries.

Over half of providers selected medical management of the third stage of labor in second trimester deliveries as first-line (53%). The second most common first-line management was expectant management, with 42% of participants reporting trial of expectant management prior to administering medications. The most commonly reported progression of methods for managing the third stage of labor for second trimester deliveries was expectant management followed by medical management followed by manual extraction followed by surgical management (27% of providers selected this sequence; Figure 1).

**Figure 2:**
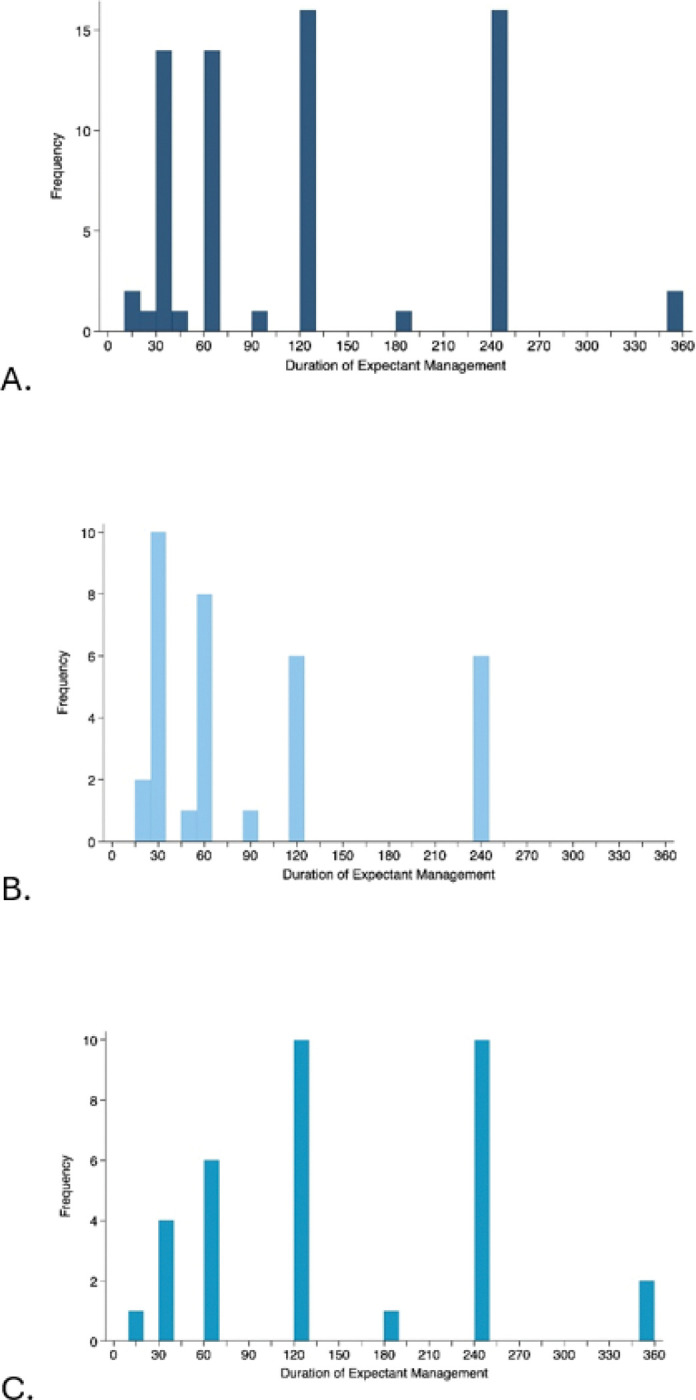
Distribution of providers responses for time allotted for expected management of the third stage of labor for second trimester deliveries among all providers (A) providers who selected expectant management as first line (B), and providers who did not select expectant management as first line (C). Providers who reported using expectant management as first line permitted shorter time allotted for expectant management compared to providers who did not select expectant management as first line (p<0.001).

Overall, providers reported a wide range of time allotted for expectant management (median 120 minutes; IQR 30, 240 minutes). Providers who selected expected management as the first-line method of management reported shorter times allotted for expectant management (median 60; IQR 30, 120 minutes) compared to providers who did not select expectant management as first line (median 120; IQR 60, 240 minutes, Wilcoxon rank-sum p<0.001, Figure 2).

There were no significant differences in selection of first-line management method by provider type, level of training, duration of practice, or practice setting. Among providers who reported that their hospital had a protocol for managing the third stage of labor for second trimester deliveries (n=35), 54% selected medical management as first-line method, 43% selected expectant management as first-line, and 3% selected surgical management as first-line.

Preferred first-line agent for medical management of the third stage of labor was most commonly oxytocin (56%), followed by misoprostol (31%), intramuscular 15-methyl prostaglandin F2a (11%), and methylergometrine (2%). Of the providers who selected oxytocin as first-line, the most common preference for a second-line medication was misoprostol (54%), then methylergometrine (18%), then intramuscular 15-methyl prostaglandin F2a (10%; Table 2). Nine percent of providers reported they would not use any medication apart from oxytocin for medical management of the third stage and 30% of providers reported they would not use any medications apart from oxytocin and misoprostol. Half of providers reported they would not use intramuscular 15-methyl prostaglandin F2a to manage the third stage of labor in a second trimester delivery (48%).

The three factors reported as the primary influence for selecting method of management were as follows: learned practice from a mentor (32.7%), scientific literature or society guidelines (26.3%), and clinical scenario (24.8%).

## Discussion

Our study sheds light on the heterogeneity of practice patterns in management of the third stage of labor following second trimester deliveries. In a group of over one hundred providers who routinely manage second trimester deliveries across the United States, preferences for management and sequence of medication administration and procedural intervention varied widely as did time permitted for expected management and medication selection.

For second trimester deliveries, there is no established consensus on the appropriate time interval to permit expectant management for placental delivery in a medically stable patient before medical or surgical intervention should be pursued. The few existing studies comparing expectant and medical management vary from supporting medical intervention immediately following delivery^1^ through over 30 minutes after delivery.^5,6,10^ The only formal recommendation for managing the third stage of labor in second trimester deliveries comes from the Society for Family Planning. These guidelines refer specifically to second trimester abortions; there are no societal guidelines for best practices in managing the third stage in second trimester deliveries following a live birth. Our survey did not specify the clinical scenario proceeding the third stage of labor given that the special considerations for placenta delivery and the risk of retained products exists for all second trimester deliveries. That said, none of the providers who responded to our survey reported that they would permit expectant management for up to four hours as recommended by the guidelines put forth by the Society for Family Planning.

Preferences for medical management also varied widely among respondents. A prospective randomized trial demonstrated 10 units of intramuscular oxytocin to have superior efficacy in leading to spontaneous placental expulsion and decreasing short term postpartum blood loss as compared to oral misoprostol or placebo.^1^ This study is referenced in the Society for Family Planning’s Clinical recommendation for oxytocin administration to aid placental delivery.^3^ In our survey, most participants selected oxytocin as a first choice in medication management, consistent with these recommendations. Two studies have highlighted the effectiveness of intramuscular 15-methyl prostaglandin F2a for the third stage of labor in second trimester deliveries: one awas randomized control trial comparing use of intramuscular 15-methyl prostaglandin F2a vs normal saline^5^ and one was a retrospective study comparing use of intramuscular 15-methyl prostaglandin F2a to rectal misoprostol.^6^ In these studies, intramuscular 15-methyl prostaglandin F2a was found to be more effective than its counterpart at causing spontaneous delivery of the placenta. Rectal misoprostol has not been shown to be effective at shortening time to placenta delivery or decreasing maternal morbidity related to delivery. ^8,10^ Despite the evidence, misoprostol was much more commonly selected as the second-line agent for managing the third stage of labor among survey respondents and half of respondents reported they would not use intramuscular 15-methyl prostaglandin F2a for management of the third stage of labor in a second trimester delivery.

One limitation of our survey is that participants were required to report a sequence in which they would pursue expectant, medical, or procedural management of the third stage of labor. Thus, the employment of multiple management strategies simultaneously may not have been captured. Additionally, the majority of respondents were generalist OB/Gyns practicing at academic hospitals in urban settings so practices in rural or community settings may be underrepresented. This may, however, be a result of the majority of second trimester deliveries occurring in urban, academic settings, given that these patients are frequently transferred if presenting to community hospitals. Lastly, our survey did not make the distinction between managing the third stage of labor in a second trimester abortion, spontaneous preterm labor, inductions for fetal demise, or medically indicated inductions leading to live births. While this differentiation was deliberately omitted, it is possible that management preferences may differ in these clinical scenarios.

## Conclusions

Overall, the wide variation in management patterns reported by our participants reflects the paucity of evidence and clinical guidelines for optimal management of the third stage of labor in second trimester deliveries. This survey highlights the need for more robust comparative trials to inform how to best care for these patients.

## Figures and Tables

**Table 1.** Characteristics of providers (N=113)

	N	%
Provider Type		
OBGYN, Generalist	92	81.4
Maternal Fetal Medicine Specialist	13	11.5
Certified Nurse Midwife	8	7.1
Level of Training		
Training complete	78	69.0
Fellow	6	5.3
Resident	29	25.7
Years of Practice		
1–4	33	29.2
5–9	23	20.4
10–14	29	25.6
15–19	10	8.8
20+	18	15.9
Delivery Setting		
Academic hospital	103	91.2
Community hospital	9	8.0
Missing	1	0.9
Practice Setting		
Urban area (population 50,000+)	107	94.7
Urban cluster (population 2,500 to 50,000)	6	5.3
Rural (population <2,500)	0	0
Geographical area*		
Northeast	17	15.0
South	36	31.9
Midwest	31	27.4
West	29	25.6
Population served		
Primarily uninsured/publicly insured	45	39.8
Primarily privately insured	16	14.2
Mixed	52	46.0

* Northeast (CT, MA, ME, NH, NJ, NY, PA, RI, VT), South (AL, AR, DC, DE, FL, GA, KY, LA, MD, MS, NC, OK, SC, TN, TX, VA, WV), Midwest (IA, IL, IN, KS, MI, MO, MN, ND, NE, OH, SD, WI), West (AK, AZ, CA, CO, HI, ID, MT, NM, NV, OR, UT, WA, WY)

**Table 2.** Characteristics of providers (N=113)

	First-Line	Second-Line*				
	N (%)	Oxytocin N (%)	Carboprost N (%)	Methylergometrine N (%)	Misoprostol N (%)	None† N (%)
Oxytocin	59 (55.7)		6 (10.2)	11 (18.6)	32 (54.2)	10 (17.0)
Carboprost	12 (11.3)	3 (25.0)		1 (8.3)	6 (50.0)	2 (16.7)
Methylergometrine	2 (1.9)	1 (50.0)	0 (0.0)		0 (0.0)	1 (50.)
Misoprostol	33 (31.1)	19 (57.6)	7 (21.2)	3 (9.1)		4 (12.1)

* Proportions are by row total;

† Provider indicated they would not try a second medication and would instead move to next method of management

## Data Availability

The datasets used and/or analysed during the current study are available from the corresponding author on reasonable request.
